# Flooding and its impacts on Nkondo community in Rundu, Kavango east region of Namibia, 1950s

**DOI:** 10.4102/jamba.v8i2.168

**Published:** 2016-01-13

**Authors:** Kletus M. Likuwa

**Affiliations:** 1 Multidisciplinary Research Centre, University of Namibia, Namibia

## Abstract

This is a study on flooding and its impact on the Nkondo community in Rundu, in the Kavango area of Namibia. It draws from archival sources at the National Archives of Namibia. Whilst archival documents provide an idea of what and how colonial officials thought of and related to the colonial subjects, they cannot represent the feelings, beliefs and interpersonal relationships of the ordinary people. This article thus made use of oral interviewing, not as a means to fill the gap but as an alternative to exploring memories of former Nkondo residents about the 1950s flood and its impact. Interviews were carried out in 2004 and 2005 when 14 people were interviewed for the histories of forced removals in Rundu, but only five are used for this article as they specifically speak to the story of flooding. Interviewees were chosen through referrals from the headmen of the surrounding villages of Rundu. Interviewees were asked questions that provide a chronological representation of a case study of forced relocations in Rundu. The article is an important historical piece that draws on unique oral-history regarding flooding and its impact. Furthermore, it is a story about power, politics and colonial dynamics and forced relocation using flooding as a pretext. The article indicates how colonial authorities made use of this benevolent excuse of a natural disaster to compel people to move permanently to new areas so as to fulfil the colonial administration’s political agenda of security and control over the population. The article indicates that flood-prone communities may fear relocating permanently due to cultural, social and economic factors. Thus, the government should not use force to relocate communities but should address communities’ fears and provide them with support in relocated areas.

## Introduction

This article relates the historical experiences of Nkondo community’s relocation due to a flood in the 1950s in Rundu in Kavango in the east of Namibia. Nkondo is the name of the village that existed near Rundu. Its date of origin is unclear. It came to be occupied mainly by the Nyemba^1^ who usually came as visitors to relatives on the Namibian side of Kavango. It seems that Nkondo village consisted of mainly of people from the Nyemba ethnic group and a scant number of Kavango people^2^ who consisted of both males and females, young and old. Nkondo is a Nyemba word that means ‘floodplains’. It was subject to cool weather as it was situated in the flood plains along the Kavango River. Nkondo was neighboured by villages such as the following: Savanyime, Nkunki, Ncwa, Mangarangandja, Sarusungu, Kanonga, Rupoworo, Uvhungu-Vhungu and Mutwarantja. Except for Kanonga village which was equally situated in a flood-plain area, all other neighbouring villages were situated on high grounds.

In the social and economic set up of Nkondo village, children and elders had separate duties. Children had various duties, but the duties were divided on the basis of gender although these overlapped at times. The girls worked in the fields, planting crops and clearing out all the bushes in the fields. They also caught fish with their fishing baskets at the river. They had to cook at home and pound Mahangu (which is a local staple cereal) to be used for cooking porridge. They had to go and collect water from the river and also looked after small children whom they usually carried on their backs. They also, like boys, collected firewood. Although boys and girls at times played together in games like playing house, this was the exception because, in most cases, boys and girls played separately. At the river, girls had separate swimming places from boys. The boys had the task of going to the river to fish. They had to look after cattle and make sure that they did not lose any of the livestock. The boys would go to the river to catch fish and cut reeds for making mats. They made bows and arrows and also helped the elders with iron smelting from which they produced knives, axes, et cetera. They helped elders to cut down trees in the forest for building homesteads and kraals. A contract-labour system also played an important role in generating additional income for many Black communities in Rundu in Namibia. The older boys were expected by their parents to become contract labourers on farms and mines in the central and southern part of Namibia so that they could bring back money to be used in paying the ‘trust-fund’ tax which every male person in Kavango over the age of 18 was compelled to pay by law. The money also served as an additional income to the household (NAR 1937:303–310; also Likuwa 2001).

There are clear signs of social control in the community, separating boys and girls from men and women. This control meant, for example, that certain social rules applied to children, especially with regard to alcohol consumption and the issue of marriage. Boys and girls would meet in the presence of their parents at the traditional dances that were usually organised for the community of Nkondo on weekend nights. Children were not allowed to drink alcohol; this was strictly reserved for the elders. Children received traditional education at ‘Shinyanga’ which was a place at the homestead section of the household head where children sat around a fire at night to be told stories, riddles and the history of the families and tribe. It was expected of the children to be married off by their parents and not to make decisions themselves. Children would inform their parents of their preferences, and the parents of the boy would go and offer a proposal to the parents of the girl. It was up to the parents of the girl to accept the proposal for marriage on behalf of their daughters or to refuse it. The elders of Nkondo village, like those of surrounding villages, worked in the fields. Very few men were employed at Rundu since jobs were scarce. The few who did work walked to work in the mornings and returned in the evenings. Men managed homesteads to see to it that there was food for their family. They went hunting for animals in the forest. They also smelted iron to make metal objects like knives and axes and to build homesteads. Women worked in the fields and did all domestic work. The women of Nkondo brewed a traditional wine known as ‘Kashipembe’ which they sold for one cent a bottle to the returning migrant labourers from ‘Dwjaini’, (a word used amongst the people of the Kavango to refer to the South-African gold mines). Although there was a school at the Roman Catholic mission in the Shambyu area, about 10 km to the east of Nkondo village where children of Nkondo could attend, most parents preferred that their children look after livestock rather than sending them to the mission school. Nkondo community got flooded in the 1950s during the South-African colonial period, and all residents were forced to vacate to higher ground. Whilst there is a growing number of studies on the flood and its impact in post-colonial Namibia, very few of these studies deal with flooding and its impact during the colonial period in Namibia. Thus, this article presents a case-study of a community’s response to flooding during the colonial period to contextualise communities’ responses to disasters and relocation in post-colonial Namibia.

## Methodology

This article used a qualitative methodological approach by drawing from both oral and archival sources. Within the National Archives of Namibia the following documents proved useful: NAR 1/1/55 was the main box where documents with the correspondence of the Natives Affairs of Rundu are located. In it, we found NAR 4, File N1/12/1, ‘Ethnologies and customs’, a letter on the system of land tenure in the Kavango, written to the secretary of South West Africa on 08 December 1929. We also found ‘Universal suffrage for Kavango’ NAR 7 by Romanus Kampungu (1970) as well as the laws of South West Africa, Vol XV1, P, Okavango Native Territory, No. 32 of 1937. Archival documents provide an idea of what and how colonial officials thought about their colonial subjects (Stoler 2002). Rassool (2004) cautions that a reliance on archival materials only can lead to the production of history from above as the colonial archives cannot represent the feelings, beliefs and interpersonal relationships of the ordinary people. Furthermore, he indicates that, in South Africa, historians have been very careful about relying on government records because of the colonial and later apartheid biases of such records (Rassool 2004). This article thus made use of oral interviewing, not as a means to fill the gap but as an alternative to exploring memories of former Nkondo residents about their experiences, their feelings and their beliefs about the 1950s flood and its impacts on their livelihoods which the archival documents cannot provide. Interviews were carried out in 2004 and 2005 by the author. A total of 14 people were interviewed for the history of forced removals in Rundu, but only 5 are used for this article as they specifically speak to the story of Nkondo village and the flood experiences. All interviewees were found in Sauyemwa Kehemu or Kaisosi settlements and Rundu, and they included G. Hairwa, L.K. Hausiku, A.M. Ihemba, R. Kambundu, B. Kambwali, S. Kahare, S. Kandere, N. Kanunga, G.N. Kaundu, S.B. Lucian, A.N. Lucian, M. Muhembo, G.L. Shikerete and A. Siyere.

Interviewees were chosen by using the snowball method, asking the headmen of the surrounding villages of Rundu who directed the interviewer to interviewees who experienced the flooding of Nkondo village. The interviewees were asked questions such as when and why the flooding occurred and what the people did in response. Answers to such questions helped to provide a chronological representation of a case study of forced relocations in Rundu from the people’s perspectives as presented in this article.

The objectives of this article are to explain why the 1950s flooding occurred, how the community experienced the flooding and the authority’s demands for relocation, what the impact of flooding were on the Nkondo community and what lessons these experiences hold for post-colonial Namibia. This article is an important historical piece that actually draws on unique oral-history interviews regarding flooding and its impact. Furthermore, it is a story about power, politics and colonial dynamics and forced relocation using flooding as a pretext. Although the story is about flooding and its impact on a community, the article indicates how colonial authorities made used of the benevolent excuse of a natural disaster to compel people to move permanently to new areas in the Mangarangandja and Sarusungu area so as to fulfil the colonial administration’s political agenda of security and control over the local population.

## Discussion

### When and why the flooding occurred

The flood which swept through and destroyed Nkondo village occurred in the 1950s. It has not been possible to establish the exact year of the occurrence of the flood and the eventual collapse of this community. In an interview, Ms B indicated that the destruction of the village of Nkondo occurred in 1954 during the time of C.E. Kruger, who was the Native Affairs Commissioner for Kavango from 1954 to 1958. Her date matches the start of the Commissioner’s first year of service in Kavango. Another interviewee, Mr D, however, gives 1958 or 1959. In the absence of official documentation about the specific date of the flood in the Kavango and given the differing date from oral sources, it can be assumed that the collapse of the community of Nkondo occurred sometime between 1954 and 1959.

Although some interviewees maintained the myth that a Mbukushu rain-making king who lived in Rundu at the time was responsible for the rain, the main point is that heavy rain fell in Rundu in that year, leading to the flood in Nkondo. The major cause of the flooding is attributed to the location of Nkondo village in a low-lying or flood-plain area where water could easily collect. Although Nkondo was indeed in a flood-plain area, it was the first time in their history that the community experienced water reaching their living space. Residents were caught off guard by the flood and were not prepared for it.

### The response of the community and the colonial authority to flooding

The respond of residents was to save their own lives by supporting each other to swim to dry land and to shift their huts and goods to high ground. Residents used ‘wato’ (canoes) which were traditional means of transportation along the river to transport people and their goods. One interviewee, Mr A, indicated the following during the interview:

‘If people were forced to move, that was the flood that made them to move out. It was a great flood that occurred so that fish would swim and enter into the huts. People moved out, their huts were falling down, what did they do? They put their hands together and picked up the huts and placed them on the boats and paddled them away. Since then, we have not seen a flood again.’ (Mr A, Male, Pensioner)

Whilst it is accepted that the flood caused the people to move, some of the interviewees argued that most of them returned to Nkondo after the water had dried out but were forced to move out by the Commissioner who threatened to beat and punish them if they remained in the flood-plain areas. Ms B explained this as follows in her interview:

‘The flood of 1954 was a very great flood. Homesteads were subsiding. Commissioner Kruger then said that we should move out of Nkondo and that anyone who would remain there would be penalised to pay a cow. But then, since the water has subsided, people did not want to move out again as they were used to living there. Kruger went in with his interpreters Paulus Munango and Makaranga and held meetings there. They said anyone who was going to remain there would pay a cow, but that before that they would be beaten with a beating stick and then made to pay a cow afterwards. People of the past feared the whites. When they heard of being beaten and forced to pay a cow that was when they moved out and came to settle at Ncwa village where the Ekongoro youth centre is now situated.’ (Ms B, Female, Pensioner)

Another interviewee, Mr C, supports the argument of being forced to move away from Nkondo village by the Commissioner after the flood had subsided:

‘People were under water, Nkondo was filled up with water, full water! They moved out and went to Mangarangandja and named that place as Mangarangandja. Flood, the whites came when the flood has gone away completely and the people were still there. The whites came and said, ‘you would suffer constantly this side, better you move away up to the highland.’ (Mr. C, Male, Pensioner)

Whilst interviews clearly point to a forced relocation, it can be argued that the Commissioner had a benevolent interest. His use of threats on the villagers can be understood as follows: It was the only way to force the move on the Nkondo villagers, who were more used to their village than any other place and were fearful that a new life somewhere else could become a miserable failure.

### The impact of flooding on Nkondo community

#### Economic impact

As the fields of Nkondo residents were situated in the flood plains, they were destroyed, leading to a loss of income because the residents could not produce food. The Nkondo residents also had to incur the cost of reconstructing homesteads in the Mangarangandja and Sarusungu area. People lost items from their homes like cups and plates which dropped off along the way or which were forgotten behind or simply destroyed by the flood.

People further incurred losses when traditional beer was spilled during the moving process. Some people’s huts were destroyed during the transportation process, and those who had built brick houses had to dismantle them. The new areas to which they were relocated were still full of wild animals like lions, elephants and wolves, which caused problems with their livestock, amongst others. As such, the new settlements on the high ground were therefore not favourable spaces for settlement at the time.

#### Social impact

Flood waters filled the entire village, destroyed homesteads and left occupants homeless. Since people had to swim through the water, some contracted diseases from contaminated water. Since the Kavango area is a malaria area, one big impact of uncontrolled water over a larger area was that mosquitos found a large breeding space which added to an increase in the occurrence of malaria amongst the population.

Residents of Nkondo stored their food in constructions next to their homesteads. This meant that the flood destroyed the traditional food storage, which led to food shortages and eventually resulted in famine amongst residents.

The residents of Nkondo village, like other communities in the Kavango, used to bury the head of a household in his cattle kraal which was usually situated behind his homestead. (See Kampungu 1970) This meant that the grave yards of the ancestors were usually situated at the homestead. The flood washed away ancestral graves, which meant that the flooding of ancestral graves and the eventual separation of the ancestral graves from their living loved ones had a psychological impact on the survivors. It was traumatic for the survivors to be separated from the ancestral graves. The survivors of the flood came to settle in the village of Ncwa, specifically in a settlement that was known as Mangarangandja [senseless loud noise] where many Nyemba ethnic groups from Angola were settled. Most of them, however, went to Sarusungu village which at the time was a sparsely populated village. Spatial change was a problem faced by almost all the relocated people. In the case of South Africa, for example, De Wet (1995) argues that, in as much as resettlement involves the movement of people from one place to another, it brings about change in the spatial setting or context in which people find themselves and to which they have to adapt. This change in spatial setting has physical, socio-economic and political implications. It is against the above context that the flooding, the eventual relocation and its effects on the community of Nkondo in Rundu are to be understood. It brought about problems associated with spatial change. In the new areas, some people from Nkondo experienced the problem of suitable space. To some of them, it was not an easy task to locate good enough space for a new settlement as the spaces were fewer and smaller than in the Nkondo area. As Mr D explained during his interview:

‘We did not like to live closely with other people. When we came we found that people have already built all over, there was no space for us to settle again, it was therefore better that we had to go and look for our own space somewhere where we could live.’ (Mr D, Male, Pensioner)

In the new areas, living space became limited and smaller than in their previous areas because there were already many people in the resettlement areas, resulting in competition for the acquisition of space. The forest land further south of the areas of Mangarangandja and Sarusungu formed part of their new subsistence-farming fields but was far away from the river.

In an example comparable to that of Nkondo, residents from Nyanga in the Eastern Cape in South Africa, too, could hardly find any good space in their relocated areas as everybody wanted a good site that was as close as possible to wood and water. Many people therefore found the sites that they wanted already occupied (De Wet 1995:87). It is true that the physical aspect of any move would largely circumscribe the degree to which people are able to preserve their old way of life. De Wet (1995) explains:

Factors such as such as whether people are close enough to their old home areas to be able to keep up contacts, or whether the area has been transformed, whether they are in an area which is topographically, agriculturally and climatically similar or different and whether they would have to find new ways of making a livelihood, will influence the way in which people will seek to organize themselves in their new situation. (p. 10)

There were other social effects of flooding and relocation, but these were not very serious. The people from Nkondo, for example, did not remain together but were scattered amongst the people of the communities of Mangarangandja and Sarusungu. It was, therefore, not possible to keep old bonds with their former neighbours from Nkondo village in the new areas as they all had to fit in an already occupied space. They were, however, able to maintain many of their traditional practices in the new areas. The people from Nkondo continued to practice their tradition of cultural performances at night in the new areas. They also maintained the practice of arranged marriage for their children. Children still had delegated domestic tasks or responsibilities according to their gender. This was so because the people they found in the new areas were in most senses similar to them, culturally and linguistically, and it was therefore no very difficult for the people of Nkondo to fit into their new spatial setting. According to De Wet (1995), the greater the change, the greater the stress:

The kind and degree of spatial change that people undergo seems to hold part of the key to a greater understanding of the stress they undergo in resettlement … the greater the physical and or social modification, the greater the stress. (p. 10)

We can only assume here that, because the Nkondo community was not relocated very far from their former area and because their new location was related to their former neighbouring villages, the social and psychological effect of the move was minimal. This is, however, an assumption as it was not entirely possible in this research to determine the extent of psychological effect on the people of Nkondo except to say that many were unhappy to be separated from their ancestral graveyards.

The relocation of Nkondo had various social consequences. When the children at the school hostel of Shambyu mission returned home, they found that their homesteads no longer existed, and they had to search for their parents in the new relocated area of Mangarangandja and Sarusungu. In a personal interview, Ms B indicated that life in Mangarangandja was not good compared to Nkondo because they came to suffer water shortages. She said that many people in the relocated areas, mainly women, would line up for water behind the water point and soon began to argue with each other. They even went as far as labelling each other as witches, and this contributed to the breakdown in neighbourliness and cooperation.

People found that, in the new sites, they were living in crowded spaces, and it was difficult to own anything compared to what they were able to have along the riverside villages. The practice of ‘Shinyanga’, which was families’ evening gathering around the fire for traditional educational purposes, common in Nkondo, began to disappear because children in Mangarangandja started moving around at night at shebeens that mushroomed. The old activities of night-time traditional dances with drum beatings were slowly but surely being replaced by the all-night disco dances at shebeens in Mangarangandja. Later, as Ms B explains in an interview, ‘When elders tried to admonish children, they became obstinate and would no longer listen.’ People became dependent for survival mainly on money rather than on farming because they settled in what were formerly their ploughing fields, and therefore, space for bigger fields became very limited.

In his interview, Mr E gave a clear description of life in Mangarangandja after the residents from Nkondo came to join the settlement:

‘Mangarangandja changed drastically as an increase in population reduced the space for residential areas. It was no longer a favourite place to raise livestock or to be a subsistence farmer, and most people by then depended on the cash economy. Mangarangandja was the area in which our colleagues who came from across [*Angola*] came to settle and gave it that name. And now, because of other people who use to come from that other side [*Angola*], Mangarangandja became a very ‘hard’ [*problematic*] village. Many young men who were found there were pilgrims. It came to happen that many cultures came to exist in it. There were cultures of Umbundu, Mbukushu, Kwangali and Gciriku. All the people came to meet there. If you came there, you would find the ‘I will give you one blow’ people, or you would also find the ‘I will kill you’ people. It therefore came to happen that when young men clashed with each other in the afternoons, the coward ones would run away. Mangarangandja became Mangarangandja. People were rough. The boys who lived there no longer behaved well. By then, we were also grown-ups now and we would also go into Mangarangandja. Ah, no, because we were born there and were known it was not a problem, we use to pass through, it was only for those who came from far and entered the area, yes. In the beginning Mangarangandja was peaceful. There were only activities and peace. But where it ended, it was very rough because there were many people and single women. So, when Magayisa [*returning contract labourers*] returned from the mines and dropped off in the compound, they would go into Mangarangandja and enjoy themselves. Most of the boys there were unemployed but needed money; those who were employed needed women, well now, stealing activities started. Life became a life that you would no longer call as a good life because by then, everybody who had ‘a broken mind’ and was rude came to Mangarangandja.’ (Mr E, Male, Pensioner)

## Conclusion

This articles indicated that the destruction of the community of Nkondo was a result of a flood that caused the whole village to be submerged under water. This gave the Commissioner a good reason to compel all the residents of Nkondo community to relocate to new areas. The relocation was based on the benevolent concern of Commissioner Kruger to prevent the community of Nkondo village from suffering from further floods. However, it shows that, even for a benevolent reason, the Commissioner could not make people move without wielding a stick and threatening to beat and fine with a cow anyone who refused to relocate. This article presents important historical data that actually draw on unique oral-history interviews. Furthermore, it is also a story about power, politics and colonial dynamics and forced relocation. Although the story is about flooding and its impacts on a community, the article indicated how colonial authorities made used of this benevolent excuse of a natural disaster to compel people to move permanently to the new areas of Mangarangandja and Sarusungu. The community of Nkondo did not re-establish itself but was dispersed amongst the people whom they found in the new areas. The article does, however, show that people were able to fit in easily in their new environment and could still maintain most of their traditional practices and customs. The central lesson from the Nkondo community’s relocation for the relocation of communities due to floods in post-colonial Namibia is that a community’s refusal to relocate from flood-plain areas to high grounds should to be understood in terms of the social, economic and cultural attachments of communities to their flood-plain villages. It should further be understood in terms of communities’ beliefs and fears of losses in relocating and settling permanently in new places. All of these concerns need to be given serious consideration and redress before and after relocating the flooded communities.

## Footnotes

1 Nyemba is the name used by Kavango ethnic groups to refer to people who came from Angola to settle in Kavango before and after the Angolan war against the Portuguese. They include groups like the Mbunda, Ngangela, Luchazi, Ngondjero and Masaka.

2 Kavango people in this article strictly refers to the following ethnic groups: Kwangali, Mbunza, Shambyu, Gciriku and Mbukushu. They are situated along the Kavango River.
